# Hydrophobic Modification of Alginate Nanofibrous Membrane by Group IV Elements Ion Crosslinking

**DOI:** 10.3390/polym18020221

**Published:** 2026-01-14

**Authors:** Takuma Yamashita, Toshihisa Tanaka

**Affiliations:** 1 Graduate School of Medicine, Science and Technology, Shinshu University, Nagano 386-8567, Japan; 22hs110h@shinshu-u.ac.jp; 2 Faculty of Textile Science and Technology, Shinshu University, Nagano 386-8567, Japan

**Keywords:** alginate nanofibrous membrane, metal ion crosslinking, hydrophobic surface, oil-water separation, dye adsorption, photocatalytic contribution

## Abstract

Hydrophobic nanofiber membranes derived from the biopolymer alginate were fabricated by electrospinning followed by metal ion crosslinking, and their potential as oil-water separation membranes was primarily investigated. Sodium alginate (SA) was co-electrospun with polyethylene glycol and subsequently crosslinked using calcium chloride and group IV metal ions (zirconium or titanium). Metal ion crosslinking changed the surface wettability of the nanofiber membranes, as confirmed by water contact angle measurements. Both zirconium- and titanium-crosslinked SA nanofiber membranes exhibited effective gravity-driven oil–water separation with complete water blocking. Although hydrophobic modification reduced direct water affinity, the resulting membranes retained residual adsorption capability toward methylene blue, indicating the presence of accessible internal polar sites. The adsorption behavior varied depending on the crosslinking ion. In addition, titanium-crosslinked membranes showed an auxiliary UV-assisted dye removal contribution under irradiation, arising from photoactive Ti species. These findings demonstrate that metal ion crosslinking provides a practical route for tuning the functional properties of alginate nanofiber membranes, with oil-water separation as the primary application and dye adsorption/photocatalysis as secondary functionalities.

## 1. Introduction

Industrial wastewater contains organic contaminants, dyes, and heavy metals that are harmful to the environment, living organisms, and human health, and therefore, purification processes are essential [1,2,3]. Among various wastewater treatment approaches, adsorbents have attracted considerable attention because of their efficiency, low cost, and operational simplicity. Numerous adsorbent materials, including zeolites, clays, and various functional membranes, have been widely investigated [4,5,6], with nanofiber membranes emerging as particularly promising candidates [7,8].

Nanofibers, which are fibers with diameters on the nanometer scale, can be fabricated by several techniques, including electrospinning, template synthesis, phase separation, and self-assembly [9,10,11]. In this study, nanofibers were prepared using electrospinning, a versatile and cost-effective technique [12]. During electrospinning, electrostatic repulsion generated under high voltage produces continuous nanofibers from polymer solutions [13,14,15]. Owing to their high porosity and large specific surface area, nanofiber membranes have shown high potential for wastewater treatment applications [16]. In particular, electrospun nanofibrous membranes have been extensively investigated for oil-water separation due to their tunable wettability, interconnected pore structures, and high permeability [17]. Therefore, nanofiber based membranes derived from polymers such as polyacrylonitrile, polyvinylidene fluoride, and polystyrene have been actively explored [18,19,20,21].

Sodium alginate (SA), a naturally derived polysaccharide extracted from brown algae, was selected as the base polymer for fabricating sustainable nanofibers. SA is the sodium salt of alginic acid, composed of *α*-*L*-guluronic acid and *β*-*D*-mannuronic acid units [22]. Each monomer contains two hydroxyl groups and one carboxyl group, imparting hydrophilicity to the polymer. Although alginic acid is insoluble in water due to strong intra- and intermolecular hydrogen bonding, water solubility increases when the carboxyl groups are neutralized by monovalent cations. SA nanofiber membranes exhibit high water solubility due to the hydrophilic nature of sodium alginate [23]. Therefore, insolubilization is required for applications in wet conditions, such as filtration and oil-water separation. In the presence of multivalent cations such as calcium, alginic acid forms “egg-box” structures through coordination with guluronic acid blocks, generating ionically crosslinked gels [22,24]. Due to its biocompatibility, biodegradability, non-toxicity, and metal adsorption capability, alginate has been extensively studied for biomedical and drug delivery applications [25,26].

Electrospinning of SA alone is challenging due to its rigid molecular chains; therefore, water soluble polymers such as polyethylene glycol (PEG) and polyvinyl alcohol (PVA) are commonly used as spinning aids [27,28]. This approach reduces the use of organic solvents and enables more environmentally conscious processing. Islam and Karim demonstrated that SA nanofibers can be obtained using PVA as a spinning aid, although a relatively high PVA content was required [29]. Wang et al. reported that SA-rich nanofibers can be fabricated at a total polymer concentration of 3 wt% by using polyethylene oxide (PEO) (SA/PEO = 90/10, wt%/wt%), and that the incorporated PEO can be removed during the crosslinking process [23].

Although Ca(II) is commonly used to stabilize SA nanofibers through ionic crosslinking, Ca-crosslinked SA remains highly hydrophilic and absorbs both water and oils, limiting its applicability in oil-water separation. To overcome this water sensitivity, various crosslinking strategies have been examined, including ionic crosslinking with calcium ions, covalent crosslinking with glutaraldehyde, and molecular modification with cysteine or cystamine to introduce reversible crosslinking sites [24,30,31,32]. However, many of these methods involve toxic reagents or time-consuming processes; glutaraldehyde, in particular, is known for its cytotoxicity [33]. Thus, ionic crosslinking provides a practical route for tuning the surface properties of SA nanofibers.

Wang et al. reported that zirconium ions convert hydrophilic alginate foams into hydrophobic materials by modifying their microstructure and reducing surface energy through strong coordination with both mannuronic acid and guluronic acid unit [34]. Recent reviews have further summarized various strategies for imparting hydrophobicity to alginate-based materials, including ionic crosslinking, composite formation, and structural modification, highlighting their potential for water treatment and membrane-based separation applications [35].

Building on this concept, in this study we investigate the use of Ca(II), Zr(IV), and Ti(IV) ions to crosslink SA nanofibers and change their hydrophilicity to hydrophobicity. The effect of different crosslinking ions on the structure and properties of SA nanofiber membranes was evaluated.

## 2. Materials and Methods

### 2.1. Material

Sodium alginate (viscosity: 1000 cps) was purchased from Nacalai Tesque Corporation (Kyoto, Japan). Methylene blue was purchased from Chroma-Gesellschaft Schmid GmbH + Co. (Stuttgart, Germany). Polyethylene glycol (*M*_w_ = 3,500,000–4,000,000), titanium dioxide (anatase type), calcium chloride, zirconium oxychloride octahydrate (ZrOCl_2_·8H_2_O), dimethyl sulfoxide (DMSO), Triton X-405, titanium chloride solution (TiCl_4_, 16–17 wt% Ti), hexane, dichloromethane, and Sudan III were purchased from FUJIFILM Wako Pure Chemical Corporation (Osaka, Japan). All chemicals were used as received without further purification.

### 2.2. Preparation of SA Nanofiber Membranes

For the preparation of SA solutions, sodium alginate (0.27 g) and polyethylene glycol (0.03 g) were added to a conical flask containing 8.7 g of distilled water and stirred overnight. Subsequently, 1 g of DMSO and 1 wt% TritonX-405 were added, and the mixture was stirred until a homogenous solution [23].

TiO_2_ containing SA solutions were prepared by dispersing 1.0 wt% TiO_2_ nanoparticles into the SA spinning solution following the same procedure. The prepared solutions were sonicated to remove air bubbles and ensure homogeneity before electrospinning.

Electrospinning (Kato tech Co., Ltd., Kyoto, Japan) was conducted using a 10 mL syringe equipped with a 21-gauge needle. The applied voltage was maintained between 25 and 35 kV with a flow rate of 0.1–0.2 mL/min. The collector was placed 15 cm from the needle tip and rotated at 20 m/min. The spinning process was carried out at 23 °C and 20% relative humidity for 5–10 h to obtain thick nanofiber membranes.

### 2.3. Crosslinking Treatment

The prepared SA nanofiber membranes were immersed in 100 mL of 5 wt% CaCl_2_ solution for 10 min, followed by thorough washing with distilled water to remove PEG and residual Ca(II) ions. The membranes were then immersed in 100 mL of 3 wt% ZrOCl_2_·8H_2_O solution for same duration and washed again several times with distilled water [34]. The crosslinked membranes were lyophilized overnight using a freeze dryer (FD-100e, Tokyo Rikakikai Co., Ltd., Tokyo, Japan). For Ti(IV) crosslinking, a titanium chloride (TiCl_4_) solution was mixed with distilled water at a weight ratio of 1:4 under continuous stirring to prepare the crosslinking solution. Gradual addition of the TiCl_4_ solution was essential to suppress rapid heat generation associated with the hydrolysis of TiCl_4_, which produces TiO_2_ and releases heat. The crosslinking procedure was then carried out using the same immersion and washing protocol described above.

### 2.4. Characterization of Electrospun Nanofiber Membranes

The chemical structure, surface morphology, and elemental distribution of nanofiber membranes were characterized by Fourier transform infrared spectroscopy (FT-IR, FT/IR-6600, JASCO Corporation, Tokyo, Japan), scanning electron microscopy (SEM, JSM6010A, JEOL Co., Ltd., Tokyo, Japan), and energy dispersive X-ray spectroscopy (EDS). FT-IR spectra were recorded using attenuated total reflection (ATR) mode with 16 scans in the range of 4000–400 cm^−1^ at a resolution of 4 cm^−1^. For SEM observation, samples were sputter-coated with platinum using an ion sputter coater (JFC-1600, JEOL Co., Ltd., Tokyo, Japan) and observed at an accelerating voltage of 10–15 kV. Average fiber diameters were measured from SEM images using ImageJ software (ImageJ, ver. 1.53k) by analyzing 50 randomly selected fibers. Elemental distributions of Ca, Zr, and Ti were examined by EDS at 10 kV with 10 scans per sample.

### 2.5. Contact Angle Analysis

Contact angle analysis was performed on nanofiber samples with distilled water using a contact angle meter (DMs-400, Kyowa interface science Co., Ltd., Saitama, Japan). Contact angle was measured 5–10 times per sample and image analysis was performed using FAMAS software (ver. 3.5) (Kyowa interface science Co., Ltd., Saitama, Japan). Contact angle was measured by dropping 2 μL of liquids onto the samples and recording the angle at 1 s after deposition. Furthermore, the titanium containing nanofiber membranes were measured before and after UV irradiation and checked the difference in hydrophobicity. UV irradiation was conducted for 24 h in the dark room at a distance of 15 cm using a 365 nm light source.

### 2.6. Filtration Property

Filtration properties of the nanofiber membranes were evaluated using mixtures of water and organic solvents. Hexane and dichloromethane (DCM) were selected as representative organic solvents. Distilled water containing a hydrophilic dye (MB) and DCM containing oleophilic dye (SudanIII) were prepared and mixed in a beaker with a total volume of approximately 20 mL at a 1:1 volume ratio. The nanofiber membranes were mounted on a filter holder with an effective opening diameter of 7 mm, and the mixture solution was poured onto the membranes for filtration under gravity at room temperature (23 ± 2 °C) and ambient pressure. For organic solvents with densities lower than water, such as hexane, the organic solvents layer formed the upper layer in the mixture. In this case, the filter holder was slightly tilted to ensure efficient contact between the membrane surface and the upper organic layer, allowing the solvent to permeate through the membrane. In contrast, dichloromethane, which has a higher density than water, formed the lower layer and permeated directly through the membrane under gravity.

The filtration process was recorded using digital camera to visualize the separation behavior. The separation efficiency was determined from the initial oil volume and the collected oil volume after filtration.

### 2.7. Dye Adsorption Property

Dye adsorption property was analyzed with Methylene blue (MB). Calibration curve for MB was created with the absorbance of a particular wavelength (664 nm). Standard solutions of 0, 0.5, 1, 2.5, 5 ppm were prepared, and their absorbance were measured by ultraviolet-visible absorption spectrometer (UV-vis (UV-2600/2700, Shimadzu Corporation, Kyoto, Japan)). Samples of approximately 5 mg were cut and immersed in 10 mL of 5 ppm MB solutions at room temperature (pH unmodified without shaking). In this experiment, Ca-Zr-crosslinked nanofiber membranes, and Ca-Ti-crosslinked nanofiber membranes were evaluated. Dye concentrations were measured at selected time points (1–96 h) using a UV-vis spectrometer, and the adsorption kinetics of nanofiber membranes were evaluated. Pseudo-first-order and pseudo-second-order kinetic models were applied to evaluate the adsorption behavior. The adsorption capacity was evaluated from equilibrium adsorption data obtained at MB concentrations of 5–1000 ppm.

### 2.8. Photocatalytic Property

Photocatalytic activity was evaluated using MB solution under UV irradiation (wavelength 365 nm, Irradiation distance 15 cm). A 5 ppm MB solution (50 mL) was prepared as the model contaminant, and nanofiber membranes (2 × 2 cm) were immersed in the solution. The samples were divided into two groups: one irradiated with UV light for 24 h and the other kept in the dark as a non-irradiated control. After treatment, the remaining MB concentration was measured by UV-vis spectroscopy, and the photocatalytic efficiency was calculated from the difference in MB removal ratio between the irradiated and non-irradiated samples.

## 3. Results and Discussions

### 3.1. Preparation of Alginate Nanofiber Membranes

**Figure 1:**
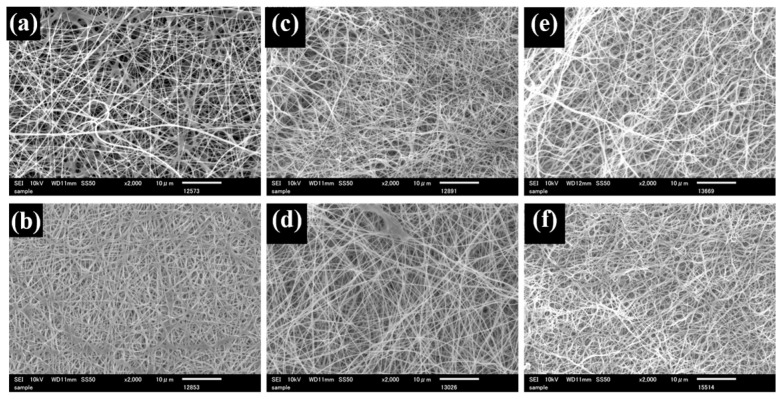
SEM images of untreated SA (**a**), Ca-SA (**b**), Ca-Zr-SA (**c**), Zr-SA (**d**), Ca-Ti-SA (**e**) and Ti-SA (**f**) nanofiber membrane.

**Figure 2:**
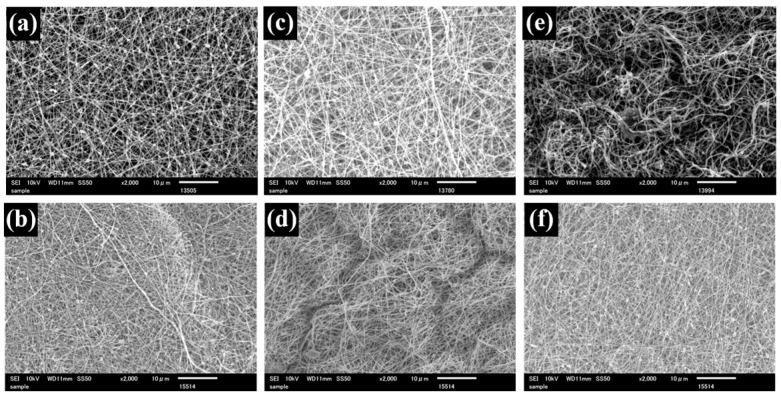
SEM images of untreated TSA (**a**), Ca-TSA (**b**), Ca-Zr-TSA (**c**), Zr-TSA (**d**), Ca-Ti-TSA (**e**), and Ti-TSA (**f**) nanofiber membrane.

Uniform SA nanofiber membranes were successfully obtained by electrospinning, as confirmed by SEM observation (Figure 1a). The average fiber diameter was 227 nm (Table 1). The smooth and continuous morphology suggests that the addition of PEG effectively improved the spinnability of SA. PEG is known to reduce solution viscosity and surface tension while suppressing excessive charge density of polyelectrolyte solutions, thereby stabilizing jet formation during electrospinning. These effects likely contributed to the formation of uniform SA nanofibers. For the TiO_2_-containing SA nanofiber (TSA), SEM images exhibited the bead-like irregularity along the fibers, which were formed by the incorporated TiO_2_ particles (Figure 2a). The average fiber diameter of TSA was 174 nm, and the TiO_2_-derived beads were approximately 444 nm in size (Table 2). The reduction in fiber diameter may be attributed to increased charge density induced by TiO_2_ particles in the spinning solution, which enhances jet stretching during electrospinning. The TiO_2_ beads are commonly observed when nanoparticles agglomerate and are subsequently embedded within nanofibers during electrospinning.

### 3.2. Two Step Ion Crosslinking Treatment

Transition metal-only crosslinking followed by lyophilization resulted in brittle membranes that fragmented easily. This brittleness was attributed to excessive crosslinking and the stresses produced during rapid drying and shrinkage [36]. The uniaxial fiber alignment caused by the rotating collector may have further contributed to the formation of oriented cracks. Therefore, Ca(II) pre-crosslinking was employed as an essential first step to stabilize the nanofiber morphology in aqueous media prior to subsequent coordination with high-valence Zr(IV) or Ti(IV) ions, which otherwise led to excessive crosslinking and membrane brittleness.

SEM images (Figure 1) show that pristine SA nanofibers exhibited uniform and smooth morphology. After Ca(II) treatment, the fiber appeared swollen (Figure 1b), consistent with the known swelling behavior of calcium alginate, which selectively interacts with G blocks in the alginate chain. In contrast, Zr(IV)-crosslinked fibers (Figure 1c) exhibited reduced diameters, indicating a higher crosslinking density arising from the ability of Zr(IV) to coordinate not only with G blocks but also with mannuronic acid units and hydroxyl groups of alginate chains [34,37]. Zr(IV) only crosslinking (Figure 1d) resulted in minimal swelling, indicating suppression of fiber expansion. These morphological trends were consistent with the average fiber diameters summarized in Table 1. Ca(II) crosslinking increased fiber diameter due to swelling, whereas Zr(IV) crosslinking, either following Ca(II) treatment or applied alone, produced smaller fiber diameters, indicating tighter polymer chain packing. This compaction is consistent with the higher charge density of Zr(IV), which promotes stronger coordination with alginate functional groups and thereby generates a relatively denser crosslinking network, as suggested in prior studies on multivalent-ion alginate gels [34,38]. In addition to the reduction in individual fiber diameter, the increased crosslinking density induced by Zr(IV) and Ti(IV) ions is considered to promote tighter fiber packing, resulting in a reduced inter-fiber spacing as observed in the SEM images. This apparent decrease in pore size is therefore attributed to morphological compaction rather than the formation of new pores.

**Figure 3:**
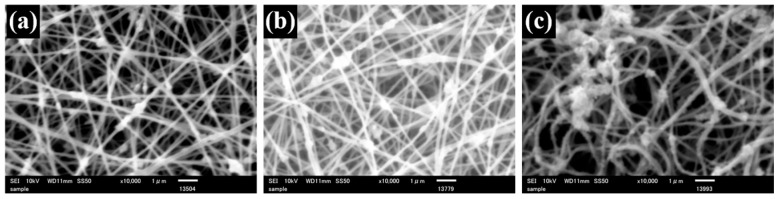
High magnification SEM images of untreated-TSA (**a**), Ca-Zr-TSA (**b**), and Ca-Ti-TSA (**c**) nanofiber membrane.

From the SEM images of TSA membranes, the bead structures remained visible after crosslinking (Figure 2 and Figure 3), while the average fiber diameter changed due to crosslinking, similar to SA nanofibers (Table 2). Transition metal ions such as Zr(IV) and Ti(IV) generally exhibit stronger coordination capability than alkaline-earth metal ions, resulting in denser crosslinked networks [39,40]. Therefore, Ti(IV)-crosslinked membranes exhibited morphological characteristics similar to Zr(IV)-crosslinked nanofibers, confirming effective ionic crosslinking and structural stabilization.

### 3.3. Structural and Chemical Characterization of Crosslinked Nanofiber Membranes

To ensure structural stability of the electrospun alginate nanofibers in aqueous media, calcium ion crosslinking was intentionally applied as a first step. This primary crosslinking stabilizes the nanofiber morphology and prevents dissolution of alginate fibers in water. Subsequent treatment with Zr(IV) or Ti(IV) ions was then employed to further modify the crosslinking structure and surface chemistry. This two-step crosslinking strategy enables morphological stabilization and functional surface modification to be decoupled, which is difficult to achieve using Zr(IV) or Ti(IV) ions alone due to their strong and rapid coordination behavior.

**Figure 4:**
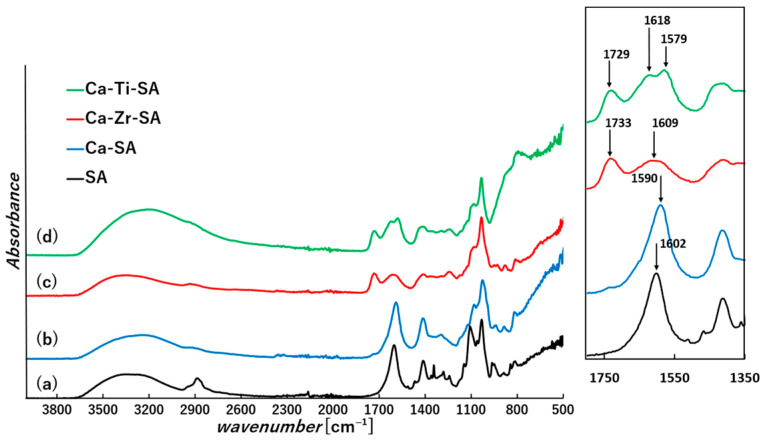
ATR-FTIR spectra (absorbance mode) of untreated (**a**), Ca-SA (**b**), Ca-Zr-SA (**c**), Ca-Ti-SA (**d**) nanofiber membranes.

The chemical structures of the nanofiber membranes were analyzed by ATR-FTIR spectroscopy (Figure 4). All ATR-FTIR spectra were recorded and displayed in absorbance mode. In the pristine SA nanofibers, characteristic peaks corresponding to the asymmetric and symmetric stretching of carboxylate groups were observed at 1600 cm^−1^ and 1405 cm^−1^, respectively, along with a broad hydroxyl stretching band at 3220 cm^−1^. Furthermore, the C-O-C stretching peak of PEG exhibited at 1100 cm^−1^. After calcium ion crosslinking, the PEG peak disappeared, and COO^−^ peaks shifted to 1595 cm^−1^ and 1419 cm^−1^, while -OH band around 3200 cm^−1^ became sharper. The shifts in the carboxylate bands can be attributed to change in the coordination environment of carboxylate groups upon interaction with Ca(II). The sharpening of the hydroxyl band suggests that the -OH groups of alginate participate in Ca(II) chelation, thereby reducing intermolecular hydrogen bonding interactions [41,42].

Furthermore, in nanofibers crosslinked with zirconium and titanium ions following calcium crosslinking, a new absorption band appeared around 1730 cm^−1^. This band is attributed to the protonation of carboxyl groups in alginic acid induced by the acidic nature of the zirconium oxide chloride solution and titanium chloride solutions [43]. In Ca-Zr-crosslinked SA nanofibers (Ca-Zr-SA), carboxyl-related peaks slightly shifted to higher wavenumbers compared to Ca-crosslinked samples, suggesting that Zr(IV) interacts with alginate carboxylate groups through coordination modes distinct from those of Ca(II). The broadening of the hydroxyl band in the Ca-Zr-SA sample further suggests the coexistence of multiple bonding modes involving Zr(IV) and hydroxyl groups. This behavior is consistent with the known coordination ability of zirconium, which can forms complexes involving both carboxyl and hydroxyl groups with partially covalent character [42].

On the other hand, Ca-Ti-crosslinked nanofibers (Ca-Ti-SA) exhibited distinct dual peaks around 1600 cm^−1^ and a broad band centered near 1400 cm^−1^. These features indicate that Ti(IV) also interact with alginate carboxylate groups through multiple coordination environments, reflecting the versatile coordination behavior of Group IV metal ions within the crosslinking network [44].

**Figure 5:**
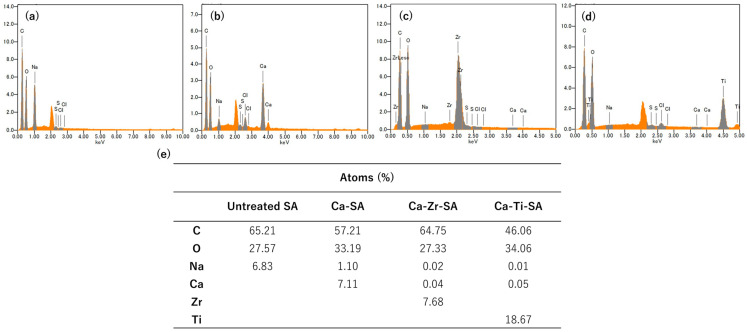
SEM-EDS result of untreated (**a**), Ca-SA (**b**), Ca-Zr-SA (**c**), Ca-Ti-SA (**d**) nanofiber membrane and atomic ratio (**e**).

The elemental distribution of the crosslinked SA nanofiber membrane was further examined using SEM-EDS (Figure 5). In pristine SA nanofiber membranes, a high sodium content was detected, whereas after Ca(II) crosslinking, both calcium and sodium were detected. The decrease in the sodium content suggests partial replacement of sodium ions by calcium ions during the initial crosslinking process. In nanofiber membrane subsequently treated with Zr(IV) and Ti(IV) ions, signal corresponding to zirconium and titanium were clearly detected, while the Ca signal significantly decreased. These results indicate that zirconium and titanium ions effectively substitute Ca(II) within the crosslinking network and act as dominant crosslinking species in the alginate matrix.

The observed decrease in apparent pore size after Zr(IV) and Ti(IV) treatment may be associated with changes in the crosslinking structure induced by multivalent metal ions, leading to tighter packing of the nanofiber network. It should be noted that this interpretation is based on morphological observations from SEM images rather than direct quantitative porosity measurements.

These spectroscopic and elemental analyses confirm that Zr(IV) and Ti(IV) ions effectively substitute Ca(II) as the dominant crosslinking species and interact strongly with alginate functional groups through multiple coordination modes. While ATR-FTIR and SEM-EDS provide clear evidence for differences in coordination environments among Ca(II)-, Zr(IV)-, and Ti(IV)-crosslinked nanofibers, the direct relationship between coordination chemistry and surface wettability cannot be fully elucidated by structural characterization alone. Therefore, the origin of hydrophobicity induced by Group IV ion crosslinking is discussed in detail in the following section, with the aid of a schematic illustration.

### 3.4. Origin of Hydrophobicity and Surface Wettability

**Figure 6:**
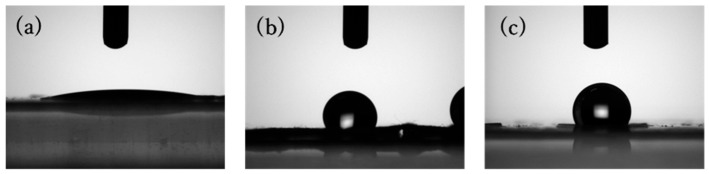
WCA images of Ca-SA (**a**), Ca-Zr-SA (**b**) and Ca-Ti-SA (**c**) nanofiber membrane.

Hydrophilic and hydrophobic properties of SA nanofiber membranes were evaluated by water contact angle (WCA) measurement. The examined samples were SA nanofiber membrane (untreated), Ca-SA, Ca-Zr-SA and Ca-Ti-SA membranes (Figure 6).

**Figure 7:**
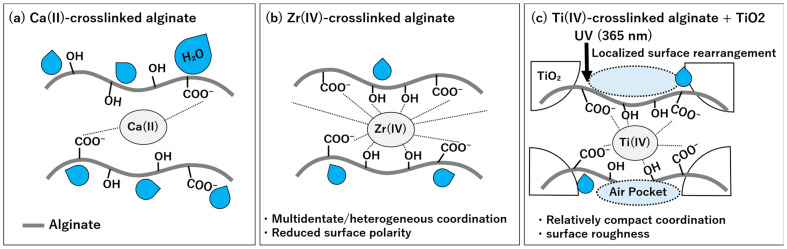
Schematic illustration of hydrophobicity induction in alginate nanofibrous membranes by Group IV ion crosslinking. (**a**) Ca(II)-crosslinked alginate nanofibers remain hydrophilic due to exposed carboxylate and hydroxyl groups. (**b**) Zr(IV) crosslinking induces hydrophobicity through multidentate coordination with alginate functional groups, which reduces surface polarity. (**c**) Ti(IV) crosslinking combined with TiO_2_ incorporation further enhances hydrophobicity via compact coordination and surface roughness, while enabling photocatalytic activity under UV irradiation.

During measurement, untreated SA nanofiber membrane dissolved in water due to its high-water solubility, confirming its strong hydrophilic nature. The Ca-SA membrane exhibited a WCA of 10.3°, which is categorized as hydrophilic because contact angles below 90° indicate hydrophilicity whereas those above 90° indicate hydrophobicity [45]. In contrast, the Ca-Zr-SA membranes exhibited WCAs of 112°, indicating a significant transition to hydrophobicity. This result is consistent with the findings of Wang et al., who reported that zirconium ion crosslinking alters the hydrophilicity of alginate foams binding hydrophilic functional groups and reducing surface polarity [34]. The same effect was observed here for SA nanofibers. Ca-Ti-SA membranes also exhibited hydrophobicity (WCA = 109°). These results indicate that Ti(IV) ions, similar to Zr(IV) ions, can induce a hydrophobic transition in alginate nanofibers. To rationalize the origin of this hydrophobic transition induced by Group IV ion crosslinking, a schematic illustration is provided in Figure 7.

As discussed in Section 3.3 (FT-IR results, Figure 4), both carboxyl and hydroxyl groups of alginates are involved in complexation with Zr(IV) and Ti(IV) ions. The high charge density of Zr(IV) and Ti(IV) ions induces coordination with multiple functional groups, which is considered to reduce the availability of hydrophilic moieties at the fiber surface by preferentially coordinating them within the polymer matrix. As a consequence, the alginate backbone becomes relatively more exposed at the surface, resulting in an apparent increase in hydrophobicity. In addition, conformational changes during crosslinking may further limit the surface accessibility of hydrophilic groups, contributing to the observed wettability transition.

**Figure 8:**
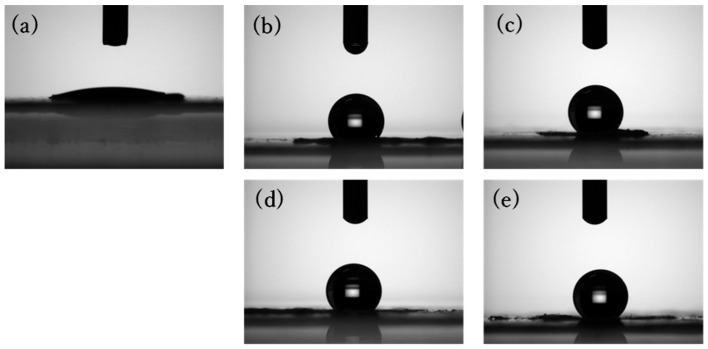
WCA images of Ca-TSA (**a**), Ca-Zr-TSA (**b**) and Ca-Ti-TSA (**c**) and after UV irradiation of Ca-Zr-TSA (**d**) and Ca-Ti-TSA (**e**) nanofiber membrane.

**Figure 9:**
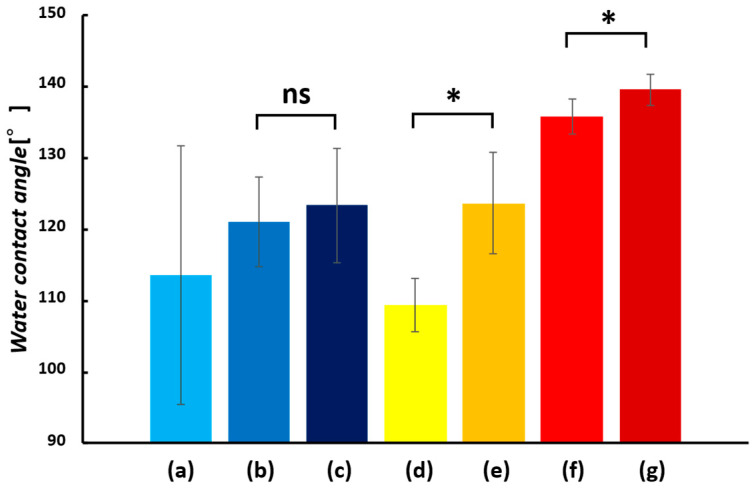
WCA result of Ca-Zr-SA (**a**), Ca-Zr-TSA (**b**), Ca-Zr-TSA UV irradiated (**c**), Ca-Ti-SA (**d**), Ca-Ti-SA UV irradiated (**e**), Ca-Ti-TSA (**f**), and Ca-Ti-TSA UV irradiated (**g**) nanofiber membrane (*n* = 10, in Ca-Ti-SA, Ca-Ti-TSA, UV−: *n* = 10, UV+: *n* = 5). Statistical significance was evaluated by Welch’s *t*-test. ns indicates no significant difference, while * indicates a significant difference (*p* < 0.05).

The WCA of TiO_2_-containing SA nanofibers (TSA) was also measured (Figure 8). The Ca-Zr-crosslinked TSA (Ca-Zr-TSA) membrane exhibited a WCA of 121°, which was higher than that of Ca-Zr-SA membrane (Figure 9). This increase can be attributed to the enhanced surface roughness due to TiO_2_ particles and fiber morphology [46]. Surface roughness promotes air retention between the water droplet and the membrane surface, enhancing apparent hydrophobicity through the Cassie-Baxter effect. Additionally, TiO_2_ particles may interact with alginate hydroxyl groups via hydrogen bonding, thereby reducing the number of hydrophilic sites directly exposed at the surface [47,48]. After Zr(IV)-crosslinking, the increased roughness is therefore considered to contribute to both air entrapment and a larger effective hydrophobic surface area, resulting in higher hydrophobicity.

The WCA of Ca-Ti-crosslinked TSA(Ca-Ti-TSA) membrane was also measured (Figure 8c). The Ca-Ti-TSA membrane exhibited a WCA of 135.8°. The Ca-Ti-TSA membrane showed the highest hydrophobicity among all samples, suggesting that TiO_2_ incorporation further enhances surface roughness and thus hydrophobicity. Both zirconium and titanium ions belong to Group IV elements and possess similar coordination behaviors. However, Ti(IV) has a smaller ionic radius (~0.061 nm) than Zr(IV) (~0.072 nm) and a slightly higher charge density, which may favor more compact coordination environments [49,50]. Such differences are likely to influence the spatial distribution of coordinated hydrophilic groups, leading to a relatively higher exposure of hydrophobic segments at the surface. It should be noted that these interpretations are based on coordination chemistry considerations rather than direct molecular-level measurements. Another possible factor is the difference in coordination number, titanium is reported to favor simpler coordination geometries with fewer hydrophilic ligands compared to zirconium, which may further contribute to the observed difference in hydrophobicity [51].

After UV irradiation, the Ti-crosslinked alginate nanofiber membranes exhibited a further increase in WCA, indicating enhanced surface hydrophobicity (Figure 9). This change is interpreted as a surface-related effect associated with photoactivation of TiO_2_ nanoparticles and Ti(IV) crosslinking sites, which may induce localized surface rearrangement rather than bulk chemical degradation. Such photoinduced effects could reduce the accessibility of surface -OH and -COOH groups, resulting in an apparent increase in hydrophobicity [52]. It should be emphasized that the present study focuses on elucidating the origin of hydrophobicity rather than quantitatively evaluating its long-term stability under continuous aqueous immersion or prolonged UV irradiation, which remains an important subject for future investigation.

### 3.5. Oil-Water Separation Performance of Hydrophobic Nanofiber Membranes

In this study, the oil-water separation performance of SA nanofiber membranes crosslinked with Zr(IV) and Ti(IV) ions was evaluated. The Ca-Zr-SA and Ca-Ti-SA nanofiber membranes were selected for filtration tests due to their mechanically stable and defect-free structures after ion crosslinking. The TSA nanofiber membranes could not be used for filtration because small cracks formed on the membrane surface during processing, resulting in water leakage through the defects. This structural fragility is attributed to the presence of TiO_2_-derived bead structures and local stress concentration during drying. Therefore, TSA membranes were excluded from oil-water separation experiments, and their performance was evaluated only in terms of surface wettability.

An oil-water mixture (hexane and dichloromethane) was prepared, and the bottom layer was dyed for visualization. Filtration was performed under gravity, either with the flask in an upright position or slightly tilted to permeate the upper oil layer. The separation process confirmed that only the oil layer permeated the Ca-Zr-SA and Ca-Ti-SA membranes, while the water layer was completely retained (Video S1). The separation efficiency was calculated from the initial oil volume and the volume of oil collected after filtration (Equation (1)), although some deviation is expected due to the volatility of the organic solvent used [53]. After filtration, the oil and water clearly separated in the beaker, and the collected filtrate volume was used to determine the separation efficiency.

(1) $$R\;(\%)=\;\frac{V_{f}}{V_{0}}\times 100$$

where *R* represents the separation efficiency and *V*_f_ and *V*_0_ represent final and initial organic solvent volume, respectively.

**Figure 10:**
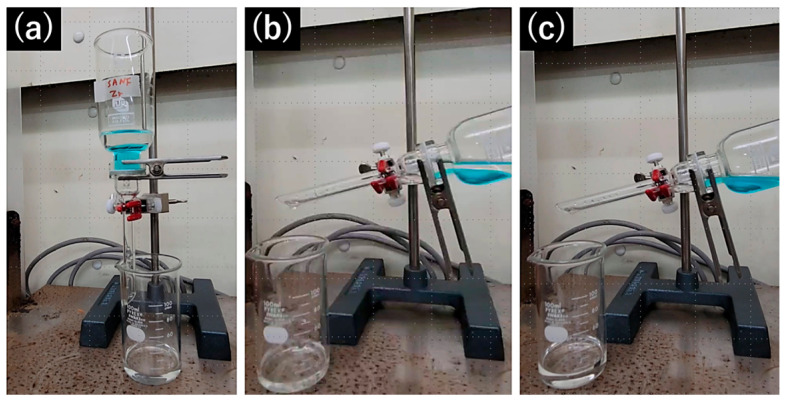
The hexane (transparent)-water (in blue) separation by Ca-Zr-SA nanofiber membrane. Retained water (**a**), tilting separator and flowing oil phase (**b**) and completely separated oil and water layers (**c**).

The Ca-Zr-SA membrane effectively exhibited oil-water separation behavior. When a hexane-water mixture was poured on to the membrane, the hexane layer readily permeated through, while the water layer was completely retained (Figure 10a). Tilting the separator enabled selective flow of the upper hexane layer through the membrane, further confirming its oil permeable and water blocking characteristics (Figure 10b,c). A similar trend was observed for dichloromethane–water mixtures. Dichloromethane, which formed the bottom layer, permeated the membranes in the same manner as hexane, while water remained fully retained on the membrane surface. Ca-Ti-SA membranes exhibited comparable separation behavior to the Ca-Zr-SA membrane. The separation efficiencies for hexane and dichloromethane were 90.3% and 90.0%, respectively, for the Ca-Zr-SA membrane. For Ca-Ti-SA membrane, the efficiencies were 91.5% and 89.3%, respectively. These results indicate that Zr(IV)- and Ti(IV)-crosslinked SA nanofiber membranes function as effective gravity-driven oil-water separation membranes, primarily due to their hydrophobic surface characteristics and interconnected fibrous structures. The present results demonstrate proof-of-concept oil-water separation without external pressure, while detailed flux evaluation and long-term reusability will be addressed in future studies.

### 3.6. Additional Functionalities: Dye Adsorption and Photocatalysis

#### 3.6.1. Dye Adsorption Behavior of Zr(IV)- and Ti(IV)-Crosslinked Membranes

##### Adsorption Isotherms

**Figure 11:**
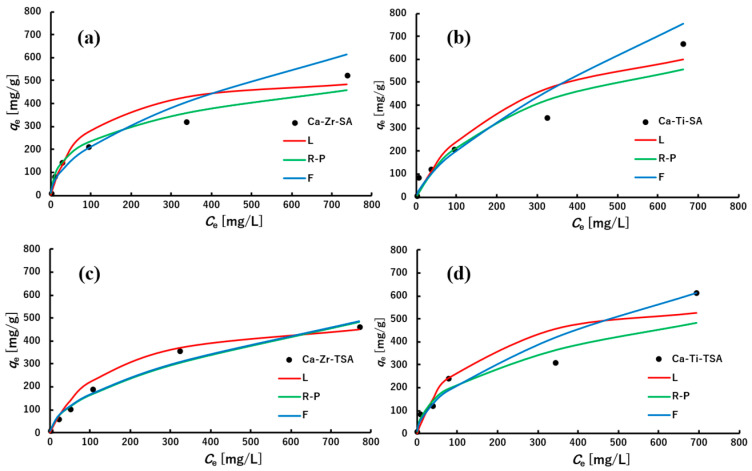
Adsorption isotherm of Ca-Zr-SA (**a**), Ca-Ti-SA (**b**) Ca-Zr-TSA (**c**), Ca-Ti-TSA (**d**), and L (Langmuir isotherm), R-P (Redlich-Peterson isotherm), F (Freundlich isotherm).

The dye adsorption performance of nanofiber membranes was evaluated using methylene blue (MB) at initial concentrations ranging from 5 to 1000 ppm. As shown in Figure 11, Ca-Zr- and Ca-Ti-crosslinked SA and TSA membranes exhibited increasing adsorption capacities with higher MB concentrations. Although these membranes exhibit hydrophobic surfaces, residual internal polar sites remain accessible to MB molecules, enabling adsorption in aqueous media. Accordingly, adsorption behavior was evaluated here as a secondary functionality to clarify whether such residual sites remain active after hydrophobic modification. To analyze the adsorption mechanism, the equilibrium data were fitted using three widely applied isotherm models, Langmuir, Freundlich, and Redlich-Peterson (Equations (2)–(4)) [54,55,56]. These models, respectively, represent ideal monolayer adsorption, heterogeneous multilayer adsorption, and a hybrid form that incorporates features of both. The R-P model showed good agreement with the experimental results across the entire concentration range, supporting the presence of mixed adsorption characteristics. The fitting parameters for all models are summarized in Table 3, and the experimental and predicted curves are shown in Figure 11.

To evaluate the goodness of fit, the sum of squares errors (SSE) was used as an error function, defined as the sum of squared differences between the experimental *q*_e_ values and the model-predicted *q*_e_ values. A smaller SSE indicates a better agreement between the isotherm model and experimental data.

Because the dataset covers a wide range of concentrations (5–1000 ppm), the experimental error is not uniform across the measurements. In particular, high concentration solutions require dilution before UV-vis analysis, which increases the relative uncertainty of *C*_e_. To minimize the effect of such uneven errors, all isotherm models were fitted using a weighted least squares approach, where the squared residuals were normalized by the square of *q*_e_ (weighted SSE). This weighting allows the fitting process to better represent low concentration regions while avoiding overemphasis of errors arising from diluted high concentration samples.


(2) $$q_{e}=\frac{q_{m}K_{L}C_{e}}{1+K_{L}C_{e}}$$

(3) $$q_{e}=\;K_{F}C_{e}^{1/n}$$

(4) $$q_{e}=\;\frac{K_{R}C_{e}}{1+\;a_{R}C_{e}^{g}}$$

The parameters *q*_m_, *K_L_*, *K_F_, n*, *K_R_*, *a_R_*, and *g* were determined from weighted non-linear regression using the experimental *q*_e_–*C*_e_ data. *q*_e_ is the equilibrium adsorption capacity (mg/g) and *C*_e_ is the equilibrium dye concentration (mg/L). *q*_m_ represents the maximum adsorption capacity, *K_L_* is the Langmuir constant and *K_F_* is the Freundlich constant, 1/*n* indicates the surface heterogeneity, and *g* expresses the deviation from ideal Langmuir behavior in the Redlich–Peterson model. Values of *g* approaching 1 indicate predominantly Langmuir-type behavior, whereas smaller values (*g* < 1) reflect stronger surface heterogeneity and increased similarity to the Freundlich model.

The results showed that the R-P model exhibited the smallest fitting errors for all nanofiber membranes, indicating that MB adsorption proceeds through a mixed mechanism rather than a single idealized process. For the Ca-Zr-SA membrane, the R-P exponent *g* was 0.71, indicating moderate surface heterogeneity. This behavior is consistent with the coordination flexibility of Zr(IV), which can generate diverse binding environments along alginate chains. In contrast, the Ca-Ti-SA membrane exhibited a higher g value (*g* = 0.90), approaching Langmuir-type behavior, suggesting a relatively more uniform distribution of accessible adsorption sites. Although Ti(IV) can form multiple hydrolyzed species in aqueous media [57], the experimental results indicate that Ti-crosslinked alginate tends to exhibit lower apparent surface heterogeneity than Zr-crosslinked alginate, which may be attributed to differences in ionic radius and coordination environments.

Introducing TiO_2_ changed the adsorption behavior. The Ca-Zr-TSA membranes exhibited the lowest *g* value (*g* = 0.47), indicating the strongest surface heterogeneity. As shown in Figure 11, the predicted R-P curve effectively reproduced the steep changes in *q*_e_ across concentrations, reflecting the existence of multiple adsorption sites associated with TiO_2_ particles and polymer-particle interfacial regions. This result suggests that TiO_2_ incorporation increases the heterogeneity of adsorption environments within the nanofiber membrane. Even in the Ca-Ti-TSA membrane, the *g* value decreased to 0.62, indicating that TiO_2_ contributes additional heterogeneous adsorption sites. Heterogeneous adsorption behavior is frequently reported for alginate-based composite adsorbents, which often exhibit Freundlich-like characteristics due to energetically diverse surface sites [58]. The Freundlich constants (1/*n* = 0.52–0.69) for all samples support these trends in surface heterogeneity.

Although the Langmuir model exhibited high theoretical capacities (*q*_m_ = 534–808 mg/g), its fitting accuracy was slightly lower than those of the R-P and Freundlich models. Zr-crosslinked alginate adsorbents are known to exhibit high MB adsorption capacity, as reported by Chen et al., which is consistent with the relatively large *q*_m_ values obtained in this study [43]. In Figure 11, the Langmuir curves show a small deviation from the experimental data at higher concentrations, indicating that monolayer adsorption alone cannot fully describe the observed behavior. Instead, the adsorption likely involves a distribution of binding energies arising from variation in metal–carboxylate coordination and the presence of TiO_2_-associated adsorption sites. Such mixed adsorption mechanisms, including electrostatic interaction, hydrogen bonding, and pore-filling effects, have also been reported for Zr-alginate adsorbents [43].

Overall, Figure 11 and Table 3 show that the MB adsorption behavior is strongly influenced by the type of crosslinking metal ion and the incorporation of TiO_2_. The R-P model provides the best description of MB adsorption, revealing a mixed mechanism involving relatively uniform metal-alginate sites and heterogeneous sites introduced by TiO_2_.

##### Adsorption Kinetics

**Figure 12:**
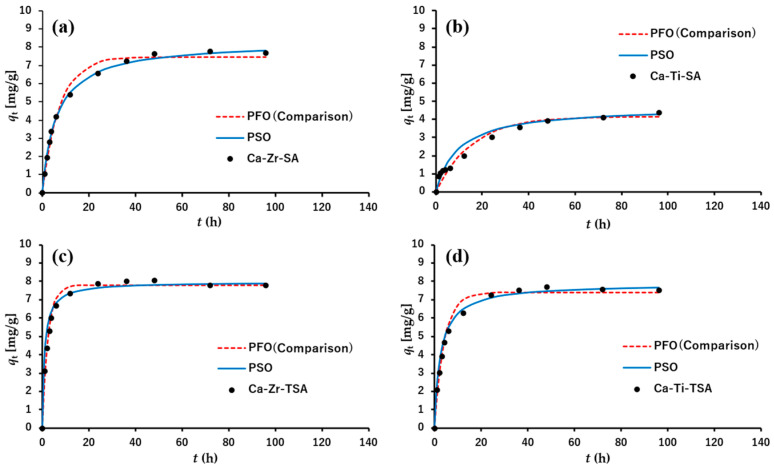
Adsorption kinetics of methylene blue on crosslinked SA nanofiber membranes. Experimental qₜ–t data are shown together with pseudo-first-order (PFO) and pseudo-second-order (PSO) model fits obtained by nonlinear regression; both models are shown for comparison under the present low-concentration condition (5 ppm). Solid and dashed lines correspond to PSO and PFO fits, respectively. (**a**) Ca-Zr-SA, (**b**) Ca-Ti-SA, (**c**) Ca-Zr-TSA, and (**d**) Ca-Ti-TSA.

The adsorption kinetics of MB were evaluated using an initial concentration of 5 ppm. The removal ratios increased during the first 24 h (Figure 12), and the final values for each membrane are summarized in Table 4. Among the tested samples, the Ca-Ti-SA membrane exhibited the lowest removal ratio. This behavior may be associated with the relatively strong coordination of Ti(IV), which can reduce the accessibility of some adsorption sites on the fiber surface. In addition, partial coverage of the nanofiber surface by TiO_2_ particles may further limit the number of readily accessible adsorption sites.

The adsorption kinetics were analyzed using pseudo-first-order (PFO) and pseudo-second-order (PSO) models. The kinetic parameters were determined by nonlinear regression of the experimental *q*_t_–*t* data. The corresponding kinetic equations are given in Equations (5) and (6) [56,59].

(5) $$\ln\left(q_{e}-q_{t}\right)=\ln\left(q_{e}\right)-\;k_{1}t$$

(6) $$\frac{t}{q_{t}}\;=\;\frac{1}{k_{2}q_{t}^{2}}+\;\frac{t}{q_{e}}$$

where *q*_e_ and *q*_t_ represent the adsorption amount (mg/g) of MB at equilibrium and at time (*t*), respectively. *k*_1_ and *k*_2_ represent the PFO and PSO rate constants, respectively.

As summarized in Table 4, both PFO and PSO models provide comparable agreement with the experimental data for all membranes, with high correlation coefficients (R^2^ > 0.99). This indicates that, under the present low-concentration condition (5 ppm), the adsorption kinetics cannot be uniquely described by a single idealized kinetic model. Rather, both models provide phenomenological descriptions of the experimental trends. Although the PSO model is often associated with chemisorption-type kinetics, the present results suggest that the kinetic models should be regarded as empirical descriptors rather than definitive mechanistic proof, and contributions from multiple adsorption interactions, such as electrostatic attraction and hydrogen bonding, cannot be excluded [60].

Overall, the kinetic analysis supports the conclusion that crosslinked SA nanofiber membranes retain accessible carboxyl and hydroxyl groups enabling MB adsorption despite their hydrophobic surface characteristics.

#### 3.6.2. Photocatalytic Contribution in Ti(IV)-Crosslinked Membranes

**Figure 13:**
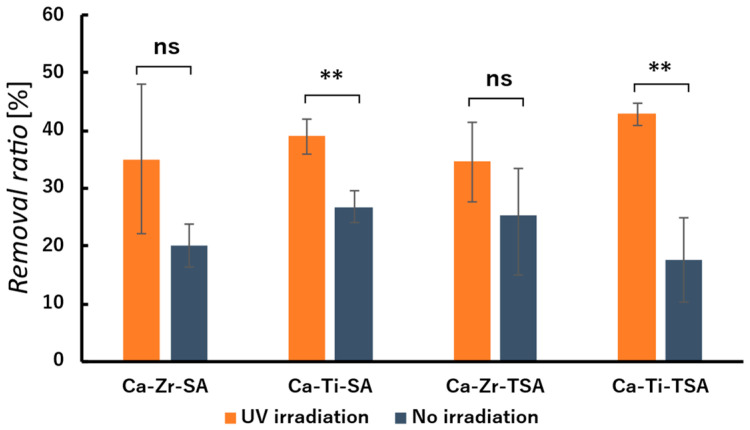
MB removal ratios of crosslinked SA nanofiber membranes under UV irradiation. Ca-Zr-SA, Ca-Ti-SA, Ca-Zr-TSA, and Ca-Ti-TSA. Statistical significance was evaluated by Welch’s *t*-test. ns indicates no significant difference, while ** indicates a significant difference (*p* < 0.01).

The photocatalytic performance of the nanofiber membranes was evaluated by UV irradiation (24 h) in MB solution (Figure 13), and the results are summarized in Table 5**.** The membranes were fully immersed in MB solution during UV irradiation, and the remaining MB concentration was quantified after 24 h. To distinguish adsorption, photolysis, and UV-assisted membrane effects, five experimental conditions were examined: blank solution under dark and UV irradiation, and membrane-containing systems under dark and UV irradiation.

Among the tested samples, the Ca-Ti-TSA nanofiber membrane exhibited the highest MB removal ratio under UV irradiation. The absolute increase in MB removal between the irradiated and non-irradiated samples was 25.3%, and the photocatalytic contribution, calculated as the relative difference between these conditions, was 59.0%. These results suggest that Ti-containing membranes provide an additional UV-assisted MB removal pathway beyond adsorption and self-photolysis. Similar behavior has been reported for TiO_2_-based thin films, in which methylene blue removal proceeds through a combined contribution of surface adsorption and UV-induced photocatalytic degradation rather than complete photocatalytic degradation [61]. A statistically significant improvement was confirmed for Ca-Ti-TSA (*p* < 0.01).

In contrast, the Ca-Zr-crosslinked nanofiber membranes also exhibited relatively high MB removal ratio; however, the difference between the irradiated and non-irradiated samples was not statistically significant (*p* > 0.05). This suggests that MB removal in Zr(IV)-crosslinked nanofiber membranes is dominated by adsorption rather than photocatalytic degradation.

Although the membranes exhibit hydrophobic surfaces, immersion in aqueous MB solution allows contact between MB molecules and accessible surface sites at the solid–liquid interface. Therefore, the enhanced MB removal observed for Ti-containing membranes under UV irradiation is attributed to the presence of photoactive Ti species, which are considered to promote MB degradation at or near the membrane surface in addition to adsorption. Consistent with this interpretation, recent studies on TiO_2_-based photocatalytic systems under UV or solar irradiation have reported that dye removal efficiency is governed by both adsorption and photocatalytic reactions, with photocatalysis providing a supplementary contribution depending on irradiation conditions [62].

Overall, these results demonstrate a functional distinction between Zr(IV)- and Ti(IV)-crosslinked nanofiber membranes: Zr-based systems remove MB predominantly through adsorption, whereas Ti-based systems exhibit combined adsorption and UV-assisted removal behavior due to the incorporation of TiO_2_ and Ti-containing crosslinking sites. Importantly, this photocatalytic contribution is regarded as a secondary functionality, complementing the primary application of the membranes in oil-water separation. In this context, photocatalytic activity is not intended to replace separation performance, but to provide an additional water-treatment-related functionality.

## 4. Conclusions

In this study, sodium alginate nanofiber membranes were fabricated by electrospinning and subsequently crosslinked using calcium and group IV element ions. Zirconium and titanium ion crosslinking effectively reduced the water solubility and enabled systematic tuning of surface wettability from hydrophilic to hydrophobic, resulting in selective oil-water separation with complete water blocking.

The crosslinked nanofiber membranes retained adsorption capability toward methylene blue, which was best described by the Redlich–Peterson model, indicating a mixed adsorption mechanism involving metal–alginate coordination sites and heterogeneous environments. For Ti-containing membranes, an additional UV-assisted dye removal was observed, whereas Zr-crosslinked membranes removed MB predominantly through adsorption.

Overall, crosslinking with group IV element ions provides an effective strategy for tuning wettability, adsorption behavior, and separation performance of alginate nanofiber membranes. The hydrophobic membranes developed in this study, demonstrate strong potential as oil-water separation membranes with supplementary dye removal functionality, offering a versatile platform for water purification applications. The long-term stability of surface wettability under repeated aqueous conditions, including UV irradiation, as well as membrane reusability, are identified as important subjects for future investigation.

## Figures and Tables

**Table 1:** Diameter of SA nanofiber and crosslinked nanofibers.

	Untreated SA	Ca-SA	Ca-Zr-SA	Zr-SA	Ca-Ti-SA	Ti-SA
Average diameter (nm)	227 ± 41	241 ± 59	168 ± 54	228 ± 55	267 ± 104	203 ± 73

**Table 2:** Fiber diameter and bead size of TSA nanofiber and crosslinked nanofibers.

	Untreated TSA	Ca-TSA	Ca-Zr-TSA	Zr-TSA	Ca-Ti-TSA	Ti-TSA
Average diameter (nm)	174 ± 54	206 ± 63	155 ± 32	161 ± 38	172 ± 46	168 ± 39
Average beads size (nm)	444 ± 139	474 ± 122	444 ± 120	460 ± 117	498 ± 123	425 ± 125

**Table 3:** Comparison of adsorption isotherm models for nanofiber membranes.

	Langmuir Model			Freundlich Model			Redlich-Peterson Model			
Sample	*q*_m_ (mg/g)	*K_L_*	R^2^	*K* *_F_*	1/*n*	R^2^	*K_R_*	*a_R_*	*g*	R^2^
Ca-Zr-SA	542.76	0.0111	0.9984	18.54	0.530	0.9992	22.27	0.317	0.712	0.9998
Ca-Ti-SA	807.91	0.0043	0.9995	8.34	0.693	0.9985	3.36	0.009	0.896	0.9996
Ca-Zr-TSA	534.23	0.0070	0.9978	14.91	0.524	0.9998	1743.38	122.135	0.470	0.9998
Ca-Ti-TSA	620.43	0.0082	0.9980	17.70	0.542	0.9993	28.90	0.720	0.617	0.9997

**Table 4:** Comparison of adsorption kinetic models for nanofiber membranes.

		Pseudo First Order			Pseudo Second Order		
Sample	*q*_e_ (Exp) (mg/g)	*q*_e_ (Cal) (mg/g)	*k*_1_	R^2^ (Non-Linear)	*q*_e_ (Cal) (mg/g)	*k*_2_	R^2^ (Non-Linear)
Ca-Zr-SA	7.770	7.440	0.134	0.9994	8.327	0.020	0.9999
Ca-Ti-SA	4.392	4.140	0.065	0.9974	4.692	0.022	0.9976
Ca-Zr-TSA	8.040	7.768	0.398	0.9997	7.948	0.126	0.9993
Ca-Ti-TSA	7.693	7.402	0.239	0.9995	7.857	0.049	0.9998

**Table 5:** MB removal by crosslinked SA nanofiber membranes under dark and UV irradiation conditions. (MB removal ratios were determined after 24 h immersion in aqueous MB solution.).

	Ca-Zr-SA	Ca-Ti-SA	Ca-Zr-TSA	Ca-Ti-TSA
Removal (UV irradiation)	35.0	39.0	34.6	42.9
Removal (Non-irradiation)	20.1	26.8	25.5	17.6
ΔRemoval (UV irra.—Non irra., (%))	14.9	12.3	9.1	25.3
Photocatalytic contribution ((UV irra.—Non irra.)/UV irra., (%))	42.6	31.4	26.7	59.0
Removal rate (mg/g)/(mg/g)	38.5	25.2	0.003	61.2

## Data Availability

Data are contained within the article and Supplementary Materials.

## Supplementary Materials

The following supporting information can be downloaded at: https://www.mdpi.com/article/10.3390/polym18020221/s1, Video S1: Oil-water separation of Ca-Zr-SA nanofiber membrane.
